# Loss of LKB1 disrupts breast epithelial cell polarity and promotes breast cancer metastasis and invasion

**DOI:** 10.1186/s13046-014-0070-0

**Published:** 2014-09-02

**Authors:** Juan Li, Jie Liu, Pingping Li, Xiaona Mao, Wenjie Li, Jin Yang, Peijun Liu

**Affiliations:** 1 Center for Translational Medicine, The First Affiliated Hospital, Xian Jiaotong University College of Medicine, 277 West Yanta Road, Xi¿an 710061, Shaanxi, People¿s Republic of China; 2 Department of Oncology, The First Affiliated Hospital, Xian Jiaotong University College of Medicine, 277 West Yanta Road, Xi¿an 710061, Shaanxi, People¿s Republic of China

**Keywords:** LKB1, Breast cancer, Cell polarity, Metastasis, Invasion

## Abstract

**Background:**

LKB1, also known as *STK11*, is a master kinase that serves as an energy metabolic sensor and is involved in cell polarity regulation. Recent studies have indicated that LKB1 is related to breast tumorigenesis and breast cancer progression. However, little work has been done on the roles of LKB1 in cell polarity and epithelial-mesenchymal transition in breast cancer. In this study, we tried to prove that loss of LKB1 disrupts breast epithelial cell polarity and causes tumor metastasis and invasion.

**Methods:**

The relationships of LKB1 expression to clinic-pathological parameters and epithelial markers E-cadherin and high-molecular-weight -cytokeratin (HMW-CK) were investigated in 80 clinical breast cancer tissue samples and their paired normal control breast tissue samples by using immunohistochemistry. Then, the LKB1 expressions in metastatic and non-metastatic breast cancer cell lines were compared. The roles of LKB1 in cell polarity and epithelial-mesenchymal transition in breast cancer were determined by using immunofluorescence, western blot assay, and cell migration and invasive assays. Finally, the non-transformed human breast cell line MCF-10A was cultured in three dimensions to further reveal the role of LKB1 in breast epithelial cell polarity maintenance.

**Results:**

Histopathological analysis showed that LKB1 expression level was significantly negatively correlated with breast cancer TNM stage, and positively correlated with ER/PR status and expression levels of E-cadherin and HMW-CK. Immunofluorescence staining showed that LKB1 was co-localized with E-cadherin at adheren junctions. In vitro analysis revealed that loss of LKB1 expression enhanced migration, invasion and the acquisition of mesenchymal phenotype, while LKB1 overexpression in MDA-MB-435 s cells, which have a low basal level of LKB1 expression, promoted the acquisition of epithelial phenotype. Finally, it was found for the first time that endogenous LKB1 knockdown resulted in abnormal cell polarity in acini formed by non-transformed breast epithelial cells grown in 3D culture.

**Conclusion:**

Our data indicated that low expression of LKB1 was significantly associated with established markers of unfavorable breast cancer prognosis, such as loss of ER/PR, E-cadherin and HMW-CK. Knockdown of endogenous LKB1 gave rise to dysregulation of cell polarity and invasive phenotype of breast cancer cells.

## Introduction

The tumor suppressor gene LKB1, also known as serine/threonine protein kinase 11 *(STK11*), encodes a serine/threonine protein kinase that has multiple cellular functions, including tumor suppression, cell cycle regulation, and promotion of apoptosis. Germ line mutations of LKB1 give rise to Peutz-Jeghers Syndrome (PJS), a rare cancer susceptibility syndrome characterized by predisposition to gastrointestinal polyposis, mucocutaneous melanin pigmentation and multi-organ cancer susceptibility [1],[2]. LKB1 serves as a master kinase responsible for phosphorylation of the conserved threonine in the catalytic domains of 14 AMPK-related protein kinases (AMPK?1, AMPK?2, BRSK1, BRSK2, NUAK1, NUAK2, QIK, QSK, SIK, MARK1, MARK2, MARK3, MARK4 and MELK) [3]-[6]. AMPK is the most important downstream target of LKB1 and functions as a cellular energy sensor. Phosphorylation of AMPK will activate TSC1/TSC2, suppress the mTOR activity and dephosphorylate mTOR effectors S6K and 4E-BP1, which are involved in regulation of protein translation initiation [4],[7]. Thus, LKB1 regulates multiple biological pathways involved in cell growth and metabolism.

LKB1 also plays crucial roles in establishment and maintenance of cell polarity. Par4, a homologue of human LKB1, has been found to control *Caenorhabditis elegans* embryonic polarity by regulating activities of anillin family scaffold proteins [8],[9]. Mutations in Par4 result in lack of germline determinant segregation, synchrony of the second embryonic division and embryonic lethality [9],[10]. Dlkb1, another homologue of LKB1 in *Drosophila melanogaster*, is required for early anterior-posterior polarity and apical-basal polarity. Cells with dlkb1 mutation show asymmetric division and abnormal spindle formation [11]. Furthermore, LKB1 helps establish and maintain cell polarity in mammals. It has been reported that loss of Lkb1 allele impairs corticofugal axon extension in telencephalic neuronal progenitors, resulting in cortical thinning and agenesis of the corpus callosum in mice. This abnormal phenotype has been traced to a defect in activation of Brsk/Sad kinases, which appear to be responsible for cytoskeletal reorganization through tau phosphorylation [12],[13]. SAD/MAPK (Par-1) kinases are other downstream targets of LKB1 and mainly expressed in the brain. LKB1 regulates the formation of axon/dendrite polarity by activating SAD/MAPK (Par-1) kinases [9],[14]. LKB1 also plays a critical role in maintenance of mammalian epithelial cell polarity. Research has shown that LKB1 can promote the formation of apical brush borders and the transport of basolateral membrane proteins in Madin Darby canine kidney (MDCK) cells [15],[16]. In addition, LKB1 can act as an upstream regulator of Par family members and increase their activities in epithelial cells of invertebrates similar to *Drosophila* and *C. elegans*[15],[17],[18]. In brief, LKB1 is an important regulator of cell polarity.

Loss of cell polarity is one of hallmarks for epithelial-mesenchymal transition (EMT) and cancer progression [19],[20]. Based on the above research, we hypothesized that loss of LKB1 may disrupt the breast epithelial cell polarity and cause tumor progression and invasion. Zhuang et al. [21] have demonstrated that overexpression of LKB1 protein reduces breast cancer microvessel density and inhibits metastasis. In the present study, our data first revealed that LKB1 expression level was significantly negatively correlated with human breast cancer TNM stage, and positively correlated with expression levels of E-cadherin and high molecular weight cytokeratin (HMW-CK) in clinical breast cancer tissue samples. In addition, the three dimensional (3D) culture system of non-transformed breast epithelial cells was used for the first time to show that loss of LKB1 disrupted the cell polarity in acini and promoted the EMT progression in breast cancer migration and metastasis.

## Materials and methods

### Patients and tissue samples

With approval by the institutional review board (IRB), a total of 80 breast cancer tissue samples and their paired normal control tissue samples were obtained from the First Affiliated Hospital of Xi¿an Jiaotong University College of Medicine and the National Engineering Center for Biochip (Shanghai, China). Clinical tumor stages were defined as stagesI, II and III according to tumor-node-metastasis (TNM) classification system. Clinicopathological characteristics of these patients from whom these tissues were obtained were presented in Table 1.

**Table 1:** Clinical profile of breast cancer patients

**Parameters**	**No. of patients (%)**
**Age(years)**	
**<60**	43(54)
**?60**	37(46)
**T-stage**	
**cT1**	21(28)
**cT2**	47(63)
**cT3-4**	7(9)
**N-stage**	
**N0**	45(60)
Nx*	30(40)
**M-stage**	
**M0**	75(100)
**M1**	0(0)
**TNM phase**	
**I**	17(21)
**II**	37(51)
**III**	21(28)
**ER status**	
**+/?**	14(20)
**++**	16(24)
**+++**	38(56)
**PR status**	
**+/?**	17(25)
**++**	15(23)
**+++**	35(52)

### Antibodies and reagents

Antibodies used in this study included anti-LKB1 (SC-32245/CST#3050), anti-E-cadherin (BD-610405), anti-?-SMA (sigma-A2547), anti-N-cadherin (BD-610920), and anti-GM130 (BD-610822). Lipofectaminet¿ 2000 (Invitrogen) was used for siRNA transfection.

### Vector and siRNA

LKB1 overexpression vector was a gift from Professor Zhijun Luo, Department of Biochemisty, Boston University School of Medicine, Boston, MA, USA. LKB1 siRNA was purchased from Invitrogen. The LKB1 siRNA sequences were as follows: LKB1-1342 sense: 5'CCG UCAAGAUCCUCAAGAAT 3'; antisense: 5'UUCUUGAGGAUCUUG ACGGTT3' and LKB1-1972 sense: 5'AAAGGGAUGCUUGAGUACG TT 3'; antisense: 5'CGUACUCAAGCAUCCCUUUTT 3'.

### Cell culture

Non-transformed breast epithelial cell line MCF-10A was obtained from ATCC (VA, USA). MCF-10A cells were cultured in DMEM/F12 (Hyclone) supplemented with 5% horse serum (Hyclone), 1% penicillin/streptomycin, 0.5 ?g/ml hydrocortisone (Sigma, H-0888), 10 ?g/ml insulin (Sigma, I-1882) and 20 ng/ml recombinant human EGF (Peprotech, 100¿15). Breast cancer cell lines MCF-7, T47D, SKBR3, and MDA-MB-435 s were cultured in DMEM (Hyclone) supplemented with 10% FBS (Hyclone). BT474 were cultured in RPMI-1640 medium (Hyclone) supplemented with 10% FBS (Hyclone). All cell cultures were maintained at 37°C in a humidified atmosphere containing 5% CO_2_.

### Immunohistochemistry

Fixed tumor tissue samples were sectioned (5 ?m), deparaffinized, rehydrated, and subjected to heat-induced antigen retrieval in EDTA Buffer (1.0 mM, pH 8.0) for 10 min in a microwave oven. Nonspecific binding sites were blocked with 10% goat serum in PBS for 30 min, and antibody against LKB1 (1:50 dilution) (SC-32245) were applied overnight at 4°C, followed by second antibodyavidin-biotin-peroxidase conjugated anti-mouse IgG (SP-9000, ZSGB-BIO 1:200 dilution) for 30 min at room temperature (RT). Proteins were visualized using 3,3?-diaminobenzidine (DAB) as the substrate.

### Immunohistochemical evaluation and statistical analysis

Imaging of immunohistochemistry (IHC) was performed using a section microscope scanner (leica MP, SCN400). The expression level of LKB1 was assessed as the percentage of the tumor cells with positive staining. The staining intensity was rated as 0 (negative), 1 (weakly positive), 2 (moderately positive), or 3 (strongly positive). Estrogen receptor/progesterone receptor (ER/PR) status was defined as low (ER/PR 0-2+), moderate (ER/PR 3-4+), and high (ER/PR 5-6+), where the numerals 0¿6 indicated the total number of ER and PR positive symbols +. The relationships of LKB1 expression to clinico-pathological characteristics and other genes were analyzed using t-test and Fisher¿s exact test. P?<?0.05 was considered statistically significant.

### Western blot assay

Cells were lysed with RIPA buffer supplemented with protease inhibitors (Roche, NJ, USA), PMSF and phosphotase inhibitors. Protein lysates were subjected to 10% SDS-PAGE, transferred to nitrocellulose membranes (Bio-Rad, CA, USA), and incubated with the indicated antibody. The reactive bands were developed by chemiluminescence with the luminol reagent (SC-2048). The blots were re-probed with GADPH antibody as a loading control.

### Immunofluorescence

MCF-7 cells were cultured on chamber slides in DMEM (Hyclone) supplemented with 10% FBS, fixed with 4% paraformaldehyde solution for 10 min at RT, washed three time with PBST and then permeabilized with 0.1% Trition X-100 for 10 min. The slides were blocked with 5% BSA and 10% horse serum in PBST for 1 h at RT and incubated with antibodies against LKB1 (1:200) (CST#3050) at 4°C overnight. After being rinsed with PBST three times, cells were incubated with secondary antibody Alexa Fluor 633 (Invitrogen #A21063) (1:200) for 1 h at RT. After being washed twice, cells were stained with 5 ?g/ml DAPI, followed by imaging with con-focal microscopy (Leica SP5II).

### Wound healing assay

Cells were seeded in 6-well plates and cultured with DMEM containing 10% FBS until cells reached sub-confluence. After removal of the culture medium, a monolayer of the sub-confluent cells was scratched with a 200 ?l pipette tip to create a wound area. The wounded monolayer were washed with PBS twice and cultured in FBS-free medium for 48 h. Cell migration into the wound area was monitored by inverted microscopy, photographed at the indicated time points, and analyzed using Leica LAS EZ software. The migration distance (distance of cell migration, mm) was calculated by subtracting the distance measured between the edges of the lesion at 48 h from the distance measured at 0 h.

### Cell invasion assay

Cell invasion experiments were performed using the Bio-Coat cell migration chamber (BD Biosciences, MA, USA), which consists of a 24-well companion plate with cell culture inserts containing a filter with 8 ?m-diameter pores. Filters were coated with basement membrane growth factor-reduced matrigel¿ (BD Biosciences, Cat#354230). Cells re-suspended in DMEM/BSA medium (3?×?10^5^ cells/500 ?l) were added to the insert (upper chamber) and DMEM containing 20% FBS was placed in the lower chamber (500 ?l per well). After incubation at 37°C for 48 h, non-invading cells were removed from the upper surface of the membrane with a cotton-tipped applicator while invading cells on the lower surface were fixed with 4% paraformaldehyde fixative and stained with crystal violet. Cells from 5 randomly chosen microscopic fields were counted and cell invasion was expressed as the number of cells per mm^2^.

### Three-dimensional cell culture

100 ?l growth factor-reduced matrigel¿ (BD Biosciences, #354230) was added to each well of a 4-well chamber slide (Nunc, Rochester, NY,177437) and incubated in a cell culture incubator for 20 min to solidify. Cells were trypsinized and re-suspended in the assay medium DMEM/F12 (5% horse serum, 0.5 ?g/ml hydrocortisone, 10 ?g/ml insulin, and 1% penicillin/streptomycin) supplemented with 2.5% matrigel and 5 ng/ml EGF. 1 ml of the cell suspension was added into the pre-coated chamber slide to generate 5000 cells/well. The chamber slide was transferred into a cell culture incubator and the assay medium (2.5% matrigel and 5 ng/ml EGF) was replaced every 4 days.

## Results

### LKBexpression level was significantly negatively correlated with breast cancer stage and positively correlated with ER/PR status

**Figure 1:**
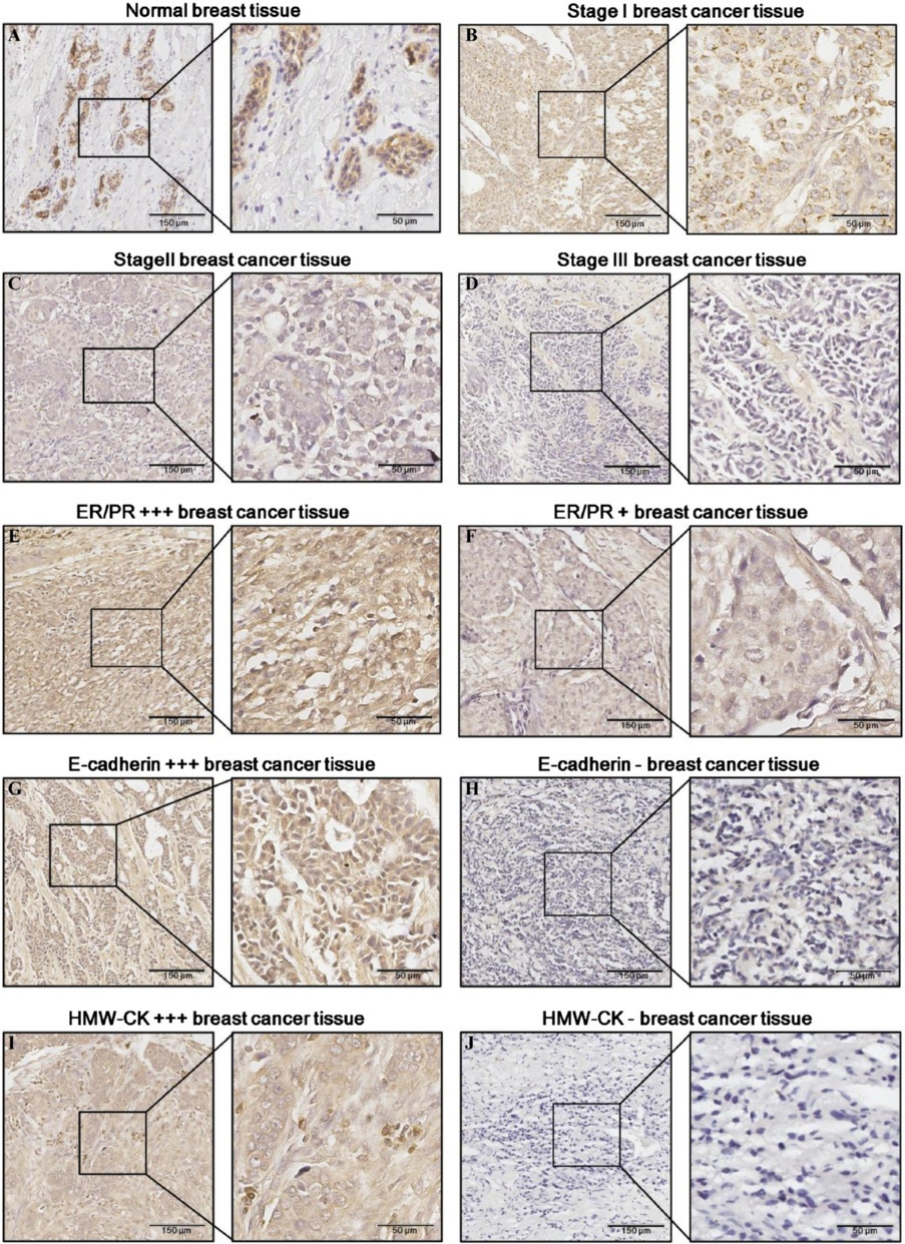
**LKB1 immunohistochemistry staining in normal mammary and breast cancer tissues.** Low-power (scale bar, 150 ?m) and high-power (scale bar, 50 ?m) photomicrographs showed **(A)** LKB1 localization in the cytoplasm of normal luminal epithelial cells, **(B)** strong LKB1 immunostaining in stageIbreast cancer tissues, **(C)** moderately positive LKB1 staining in stage IIbreast cancer tissues, **(D)** weakly positive LKB1 staining in stage III breast cancer tissues, **(E)** strong LKB1 immunostaining in ER/PR+++ breast cancer tissues, **(F)** weakly positive LKB1 immunostaining in ER/PR?+?breast cancer tissues, **(G)** strong LKB1 immunostaining in E-cadherin+++ breast cancer tissues, **(H)** negative LKB1 staining in E-cadherin breast cancer tissues, **(I)** strong LKB1 immunostaining in HMW-CK+++ breast cancer tissues, and **(J)** negative LKB1staining in HMW-CK breast cancer tissues.

**Figure 2:**
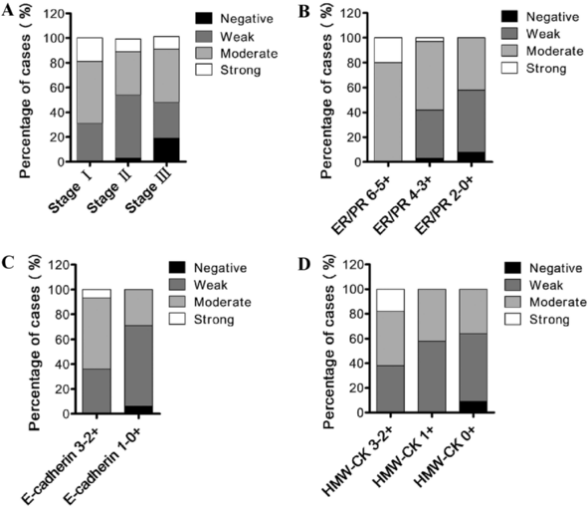
**The relationship of LKB1 expression level with cancer stage, ER/PR status and HMW-CK expression.** Distribution of breast cancer cases showing the relationship of LKB1 expression level with cancer stage (stage I/stage II/stage III) **(A)**, with **(B)**, with E-cadherin expression **(C)**, and with HMW-CK expression **(D)**.

LKB1 expression levels in normal mammary and breast cancer tissues were determined by immunohistochemistry. Normal mammary tissues showed uniform and strong cytoplasmic staining of LKB1, when compared with breast cancer tissues (Figure 1A). Statistically significant LKB1 down-regulations were detected in different stages (TNM) of breast cancer (Table 2) (Figure 1B,C,D) (Figure 2A). Moderately strong staining was observed in 69% (11 cases) of stageIbreast cancer. Forty-five percent (17 cases) of stageII breast cancer showed moderately strong LKB1 staining while 51% (19 cases) had weak staining. Four (19%) out of 21 stage III breast cancer cases were ¿completely negative¿ for LKB1 staining. In summary, stageII cases demonstrated reduced levels of LKB1 staining, when compared with stageIcases (*p*?=?0.0021), and stage III cases showed loss or reduced levels of LKB1 staining, when compared with stageIcases (*p*?=?0.0006).

**Table 2:** Association of LKB1 immunoreactivity with human breast tumor stage

**Clincal stage**	**Negative <25%**	**Weak 25%-50%**	**Moderate 50%-75%**	**Strong >75%**	**Score**	**P value**
**Stage I**		5(31)	8(50)	3(19)	30	¿
**Stage II**	1(3)	19(51)	13(36)	4(10)	57	0.0021
**Stage III**	4(19)	6(29)	9(42)	2(10)	30	0.0006

Fisher¿s exact test was performed to determine the association of LKB1 immunoreactivity with stage I, stage II or stage III breast cancer.

Interestingly, a significant correlation was found between LKB1 expression and ER/PR status in breast cancer. ER/PR positive breast cancer had an LKB1 expression level several times higher than that of ER/PR negative breast cancer (Figure 1E and F) (Figure 2B). Breast cancer cases with low or moderate ER/PR status showed reduced levels of LKB1 staining, when compared with high ER/PR cases (*p*?=?0.018) (*p*?=?0.0007). Eighty percent (20 cases) of breast cancer cases having high ER/PR status were moderately stained for LKB1, in contrast to 55% (20 cases) of moderate ER/PR cases and 42% (5 cases) of low ER/PR cases.

### LKBexpression level was positively correlated with expression levels of E-cadherin and HMW-CK in clinical breast cancer tissues

E-cadherin is an important cancer metastasis suppressor and is required for cell-cell adhesion in the epithelial tissue [22]. High-molecular-weight keratins (HMW-CK) include CK1, CK5, CK10 and CK14, which can be recognized by monoclonal antibody 34?12 we used in this study. Among these cytokeratins, CK5 and CK14 are two widely used markers for the basal epithelial phenotype and for the basal-like breast cancer, which is often associated with poor prognosis and invasion phenotype [23]. Therefore, we investigated the relationships of LKB1 to E-cadherin and HMW-CK in clinical breast cancer tissues, hoping to reveal the role of LKB1 in breast cancer progression.

Here, expression levels of E-cadherin were categorized as low (?/+) and high (++/+++). Expression levels of HMW-CK were categorized as negative (?), low (+) and high (++/+++). Significant positive correlations were detected for the first time between LKB1 intensity and metastasis suppressor E-cadherin and between LKB1 intensity and basal-like breast cancer marker HMW-CK. As shown in Table 3, 71% (22 cases) of breast carcinomas having low E-cadherin expressions showed negative or weak staining for LKB1, in contrast to 36% (10 cases) of the high expression cases (Figure 1G and H) (Figure 2C). Fifty-eight percent (7 cases) of breast carcinomas with low HMW-CK expressions showed weak staining for LKB1, and 64% (7 cases) negative HMW-CK samples showed negtive or weak staining for LKB1, as compared to 38% (6 cases) of the high HMW-CK expression cases (Figure 1I and J) (Figure 2D), indicating that LKB1 expression level was positively correlated with the expression level of HMW-CK in clinical breast cancer tissues (Figure 1I and J) (Figure 2D).

**Table 3:** Association of LKB1 immunoreactivity with clinicopathologic features of human breast cancer

**Clincal Stage**	**Negative <25%**	**Weak 25%-50%**	**Moderate 50%-75%**	**Strong >75%**	**Score**	**P value**
**ER/PR(6¿5)**			20(80)	5(20)	40	¿
**ER/PR(4¿3)**	1(3)	12(39)	17(55)	1(3)	49	0.018
**ER/PR (2¿0)**	1(8)	6(50)	5(42)		16	0.0007
**E-cadherin(++/+++)**		10(36)	16(57)	2(7)	48	¿
**E-cadherin(+/?)**	2(6)	20(65)	9(29)		38	0.0003
**HMW-CK(++/+++)**		6(38)	7(44)	3(18)	29	¿
**HMW-CK(+)**		7(58)	5(42)		17	0.0003
**HMW-CK(?)**	1(9)	6(55)	4(36)		14	< 0.0001

Fisher¿s exact test was performed to determine the association of LKB1 immunoreactivity with ER/PR, her2, E-cadherin or HMW-CK.

### LKBexpression level was decreased in metastatic breast cancer cell lines

**Figure 3:**
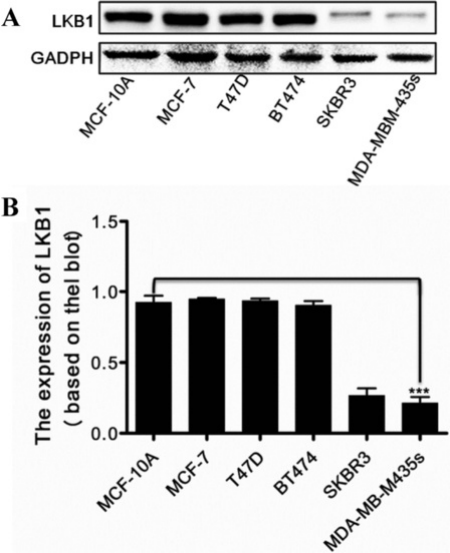
**LKB1 expressions in different breast cancer cell lines. (A)** LKB1 expressions in immortalized breast epithelial cell line MCF-10A, luminal A subtype (MCF-7, BT474 and T47D) and metastatic SKBR3 and MDA-MB-435 s. **(B)** LKB1 expressions were quantified using scanning densitometry and LKB1 expressions in breast cancer cells were calculated as ratios to the LKB1 expression in immortalized breast epithelial cell line MCF-10A.

To further determine whether LKB1 expression is associated with metastatic progression of breast cancer, we examined the expression levels of LKB1 in immortalized breast epithelial cell line MCF-10A and breast cancer cell lines, including luminal A subtype (MCF-7 and T47D), luminal B subtype BT474, and metastatic SKBR3 and MDA-MB-435 s. As shown in Figure 3, LKB1 protein levels were decreased in metastatic breast cancer cell lines. The expression levels of LKB1 in immortalized and luminal A/B subtypes were much higher than those in the metastatic cells. For instance, the LKB1 protein level in MCF-7 was approximately 3-fold higher than that in MDA-MB-435 s. This result further proved our speculation that LKB1 was involved in regulation of metastatic progression of breast cancer. Thus MCF-7 and MDA-MB-435 s cell lines were chosen to test the roles of LKB1 in breast cancer migration and invasion in the following experiments.

### LKBregulated breast cancer cell migration and invasion

**Figure 4:**
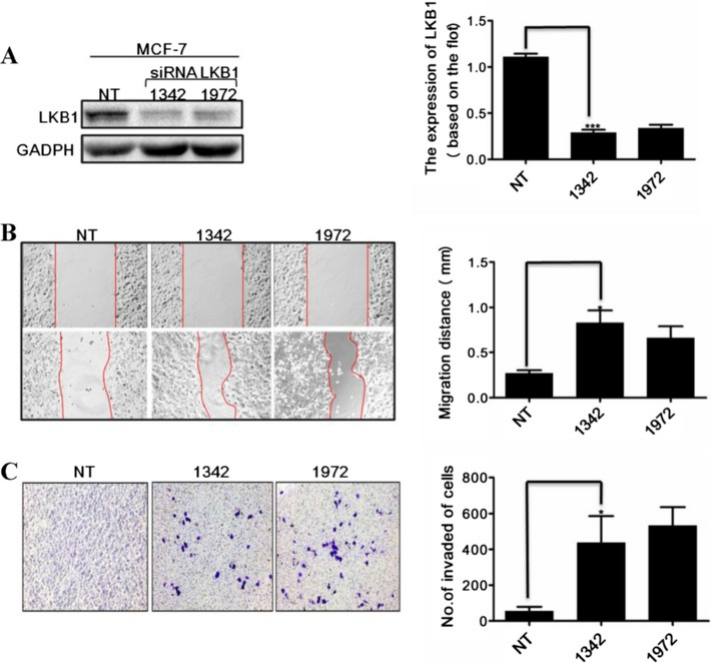
**LKB1 affected migration and invasion of breast cancer cell lines. (A)** LKB1 protein levels in control non-targeting siRNA (NT) and siLKB1 1342/1972 transfected MCF-7 cells were detected by Western blot. LKB1 knockdown efficiency was quantified using densitometry. **(B)** Loss of LKB1 increased cell migration in wound healing assay. Cell migration into the wound area was photographed at indicated time points, and the distance between the edges of the lesion was calculated as the migration distance (mm). **(C)** Loss of LKB1 increased cell invasion in matrigel transwell chambers. Migrating cells were stained and photographed. Invasion properties were expressed as the number of cells per mm^2^.

LKB1 was knocked down in LKB1-expressing MCF-7 cells by small interfering RNA (siRNA) to gain insight into the roles of LKB1 in breast cancer metastasis. The migration and invasion of LKB1 knock-down cells were assessed with monolayer wound healing assay and invasion assay. As shown in Figure 4, LKB1 knock-down MCF-7 cells showed an approximately 2-fold increase in migration and a 3-fold increase in invasion, compared with LKB1-expressing MCF-7 cells, indicating that LKB1 knockdown significantly induced the migration and invasion of MCF-7 cells.

Our results indicated that LKB1 suppressed the migration and invasion of breast cancer cells *in vitro*. This finding inspired us to speculate on the underlying mechanisms for regulation of invasion and metastasis by LKB1. LKB1 is an important regulator of cell polarity and is involved in regulation of epithelial-mesenchymal transition (EMT) [24], while EMT is a biologic process that allows epithelial cells to lose their cell polarity and cell-cell adhesion and to gain migratory and invasive properties [25]-[28]. Thus we examined whether LKB1 suppressed migration and invasion of breast cancer cells by regulating EMT and cell polarity in the following section.

### Loss of LKBup-regulated N-cadherin and ?-SMA but down- regulated E-cadherin

**Figure 5:**
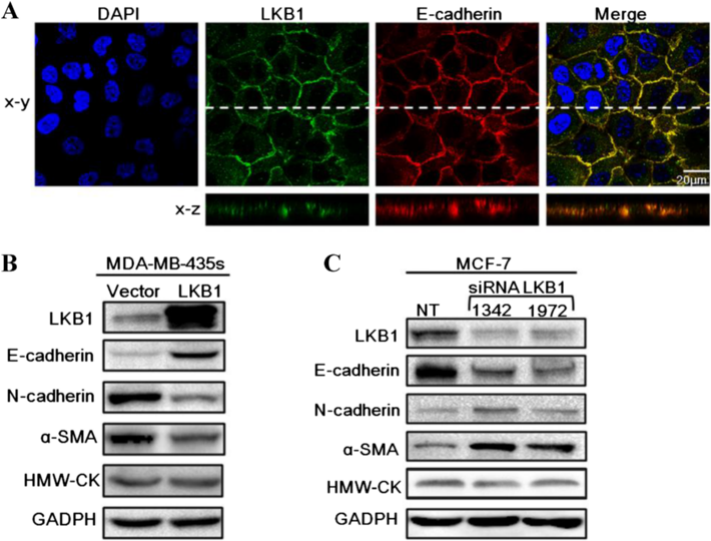
**LKB1 was localized at adheren junctions and regulated the expressions of EMT markers. (A)** MCF-10A was stained for LKB1 (green), E-cadherin (red) and DAPI (blue). **(B)** Expressions of EMT markers in control and LKB1 overexpressing MDA-MB-435 s. **(C)** MCF-7 cells were transfected with non-targeting siRNA (NT) or siLKB1(1342/1972). The protein levels of E-cadherin, N-cadherin and ?-SMA were determined by western blot.

**Figure 6:**
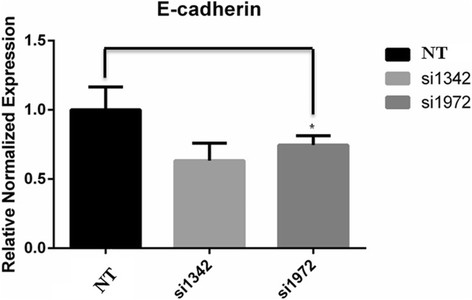
**E-cadherin mRNA expression was down-regulated in LKB1 knock-down cells.** MCF-7 cells were transfected with non-targeting siRNA (NT) or siLKB1(1342/1972). The mRNA levels of E-cadherin were detected by RT-RCR.

To investigate the function of LKB1 in regulation of cell polarity and EMT process, we first examined its location in breast epithelial cells. Immunofluorescence staining showed that endogenous LKB1 was localized at cell-cell junctions of MCF-10A cells. Z axis projections showed that LKB1 was mainly co-localized with E-cadherin at adheren junctions (Figure 5A). After that, we examined the expressions of epithelial marker E-cadherin and mesenchymal markers N-cadherin and ?-SMA in LKB1 knock-down MCF-7 cells and LKB1 over-expressing MDA-MB-435 s cells. LKB1 over-expression induced strong up-regulation of E-cadherin and significant down-regulation of N-cadherin and ?-SMA (Figure 5B). On the other hand, N-cadherin and ?-SMA were up-regulated while E-cadherin was down-regulated in LKB1 knock-down cells (Figure 5C). LKB1 knock-down induced down-regulation of E-cadherin in mRNA and protein levels (Figure 6).

### LKB1 knockdown disrupted breast acinar cell polarity in 3D culture

**Figure 7:**
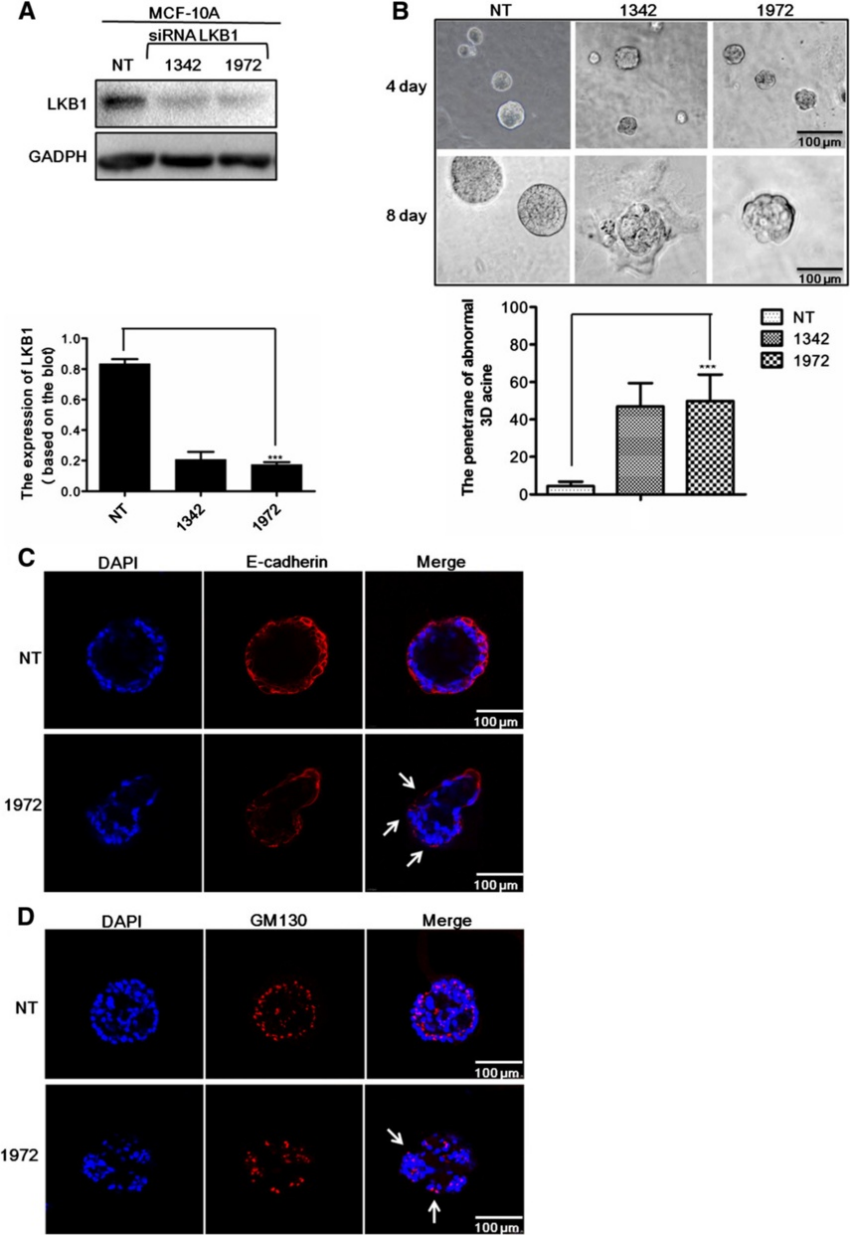
**Loss of LKB1 resulted in morphological alterations and dysregulation of cell polarity in 3D cultures. (A)** MCF-10A cells were transfected with non-targeting siRNA (NT) or siLKB1(1342/1972). The protein levels of LKB1 were detected by western blot. Efficiency of knockdown was quantified using densitometry. **(B)** Morphogenesis of MCF-10A cells plated on matrigel. Phase images of MCF-10A cells were taken on Day 4 (scale bar, 100 ?m). A single acinar structure was taken on Day 8 (scale bar, 100 ?m). **(C)** non-targeting siRNA (NT) or siLKB1(1342/1972) transfected MCF-10A cells stained with E-cadherin (red) on Day 16. **(D)** non-targeting siRNA (NT) or siLKB1(1342/1972) transfected MCF-10A cells stained with GM130 (red, Golgi marker) on Day 12.

To gain insight into the role of LKB1 in maintenance of epithelial cell polarity, we performed the 3D morphogenesis experiment using either non-targeting siRNA (NT) or siLKB1(1342/1972) transfected MCF-10A cells (Figure 7A). When MCF-10A cells were cultured in 3D reconstituted basement membrane, they reorganized and differentiated to form acinar structures, characterized by apical-basolateral polarization [29],[30]. Interestingly, siLKB1 transfected MCF-10A cells formed acini with abnormally uneven gross morphology. Fifty percent of siLKB1 transfected cells displayed irregular and rough acinar surfaces, compared to non-targeting siRNA (NT) transfected cells (Figure 7B). E-cadherin staining and *cis*-Golgi matrix protein GM130 staining [31],[32] were conducted to further reveal the morphological alterations in non-targeting siRNA and siLKB1 acini. Typical features of the siLKB1 acini included uneven and rough basolateral surface (Figure 7C) and partial loss of apical polarity due to the localization of GM130 at the basolateral surface in several MCF-10A cells (Figure 7D). Altogether, the absence of LKB1 led to gross abnormality in the acinar structure and partial loss of cell polarity of the 3D acini.

## Discussion

LKB1 is a tumor suppressor gene and loss or mutation of LKB1 has been detected in various tumors, including human breast cancer. Patients with PJS, a syndrome caused by LKB1 germline mutations, have a 54% increased risk of developing breast cancer, compared to the healthy population [21] and LKB1 is mutated in 30% of sporadic breast cancers [21],[33]-[35]. In the present study, we detected that LKB1 expression was negatively correlated with high tumor stage (TNM). We also reported a statistically significant correlation between LKB1 expression and ER/PR status. ER/PR positive tumors showed higher LKB1 expressions than ER/PR negative tumors, which agrees well with Shen et al¿s finding [33]. It has been found that LKB1 can act as a co-activator of ER? signaling and enhance ER? transactivation via its catalytic activity [36]. Contrary to our results, Singh et al. showed that LKB1 was repressed by ER? at the transcriptional level in breast cancer cell lines [37]. This discrepancy may be due to two reasons: (1) We used clinical tissue samples to study the correlation between LKB1 and ER, while Singh et al. used breast cancer cell lines. The mutual regulation of LKB1 and ER may be more complex in tissues than in cell lines. (2) Other factors or multi-layer mechanisms may exist for regulating LKB1 and ER expressions in tissues. The detailed mechanism for the mutual regulation of LKB1 and ER? is largely unknown,further studies are needed to elucidate the mechanism.

Our results showed that LKB1 expression was positively correlated with HMW-CK expression in clinical breast cancer tissues. A recent study presented by Joensuu has shown that HMW-CK5 is significantly reduced in metastasis tumor tissues [38]. And alteration of cytokeratin expression and partial loss of the normal regulation of cytokeratin expression during carcinogenesis and tumor progression have been demonstrated [39]. Together with above studies, our study indicated that alterations or partial loss of LKB1 and HMW-CK may occur simultaneously in clinical breast cancer tissues during tumor progression. To further demonstrate the positive correlation between LKB1 and HMW-CK, western blot assays were conducted to determine the expressions of LKB1 and HMW-CK in breast cancer cell lines. Unexpectedly, HMW-CK was detected in all cells and showed no difference between control, LKB1 knockdown and LKB1 overexpressing cells (Figure 5B and C). The inconsistency between clinical tumor tissues and cell lines may be that HMW-CK accumulation needed a longer time than the period of validity of transient transfection, or may be that other cells or factors in tumor tissues participated in regulating HMW-CK expression. The detailed regulatory mechanism of HMW-CK expression warrants further investigation.

LKB1 protein expression levels were highly variable among different stages of breast cancer and were potentially associated with cancer metastasis and patient outcome. It has been reported that LKB1 knockdown increases motility and invasiveness of lung cancer cells and induces expressions of several mesenchymal markers and an E-cadherin transcriptional repressor [25],[26]. Sebbagh et al. have found that LKB1 is colocalized with E-cadherin at adherins junctions in Caco-2 and MDCK II cells [24]. In this study, the colocalization of endogenous LKB1 and E-cadherin at adheren junctions was also found in breast epithelial cells. A significant correlation was detected between LKB1 intensity and E-cadherin in breast cancer tissues. E-cadherin down-regulation and N-cadherin up-regulation were detected in LKB1 knock-down cells, which indicated that loss of LKB1 expression may induce the EMT process by promoting the cadherin switch from E-cadherin to N-cadherin in MCF-7 cells. EMT is a biologic process that allows epithelial cells to lose their cell polarity and cell-cell adhesion, and to gain migratory and invasive properties [17]. Our results showed that loss of LKB1 enhanced the migration and invasion of breast cancer cells. Therefore, LKB1 may play important roles in migration and invasion of breast cancer by regulation of cell polarity.

LKB1 (*STK11/Par4/dlkb1*), as a master regulator, is needed for formation of proper epithelial architecture and cell polarity in mammals [13],[40]. LKB1 resides upstream of the Par protein network. Activation and translocation of LKB1 by STRAD-MO25 result in phosphorylation of Par1/MARK1-4. Activated Par1 in turn phosphorylates Par3, which creates binding sites for Par5 [41]-[43]. In our study, MCF-10A epithelial cell 3D culture further proved that LKB1 was necessary for formation of polarized acinar structures. Loss of LKB1 led to change in Golgi orientation and abnormal apico-basal polarity of acinar structures. However, the molecular mechanisms by which LKB1 regulates cell polarity in breast cancer remain uncertain. The 3D culture of MCF-10A epithelial cells offers the possibility to study the sequential effects of LKB1 on potential downstream polarity regulators, such as other Par homologs, Scrib/Lgl/Dlg complex and Crumbs family.

## Conclusions

Our data indicated that low expression of LKB1 was significantly associated with established markers of unfavorable breast cancer prognosis such as loss or decrease of ER/PR, E-cadherin and HMW-CK expressions. Knockdown of endogenous LKB1 gave rise to dysregulation of cell polarity and invasive phenotype of breast cancer cells. Further studies are needed to reveal the mechanisms underlying the effect of LKB1-induced cell polarity on breast cancer metastasis and invasion.

## Abbreviations

LKB1: Liver kinase B1

PJS: Peutz-Jeghers syndrome

AMPK: AMP-activated protein kinase

TNM: Tumor-node-metastasis

ER: Estrogen receptor

PR: Progesterone receptor

EMT: Epithelial-mesechymal transition

siRNA: Small interfering RNA

## Competing interests

The authors declare that they have no competing interests.

## Authors¿ contributions

J Li and J Liu performed IHC on clinical tissue samples and western blot, 3-D MCF10A cell culture, IF assay and wound healing assay. PL, XM and WL were responsible for clinical sample collection and cell handing. JY and PL were involved in the experimental design and conception, and data analysis. JL and PL wrote the manuscript. All authors read and approved the final manuscript.
