# Correction: Loss of LKB1 disrupts breast epithelial cell polarity and promotes breast cancer metastasis and invasion

**DOI:** 10.1186/s13046-025-03317-7

**Published:** 2025-02-24

**Authors:** Juan Li, Jie Liu, Pingping Li, Xiaona Mao, Wenjie Li, Jin Yang, Peijun Liu

**Affiliations:** 1 Center for Translational Medicine, The First Affiliated Hospital, Xian Jiaotong University College of Medicine, 277 West Yanta Road, Xi’an, Shaanxi 710061 People’s Republic of China; 2 Department of Oncology, The First Affiliated Hospital, Xian Jiaotong University College of Medicine, 277 West Yanta Road, Xi’an, Shaanxi 710061 People’s Republic of China

**Correction: J Exp Clin Cancer Res 33, 70 (2014)**

10.1186/s13046-014-0070-0

Following the publication of the original article [1], the authors identified errors in Figure 5c. The GAPDH protein band in Figure 4A and Figure 5C were obtained from two separate replicate experiments. However, the LKB1 protein band in both figures originated from the same experimental set. Therefore, the LKB1 band in Figure 5C requires replacement.

- Fig. 5c: LKB1 band needs replacement.

The corrected figures are provided below:

The corrections do not affect the overall results, discussion, or conclusion of the article.

**Incorrect Figure 5**

**Fig. 5:**
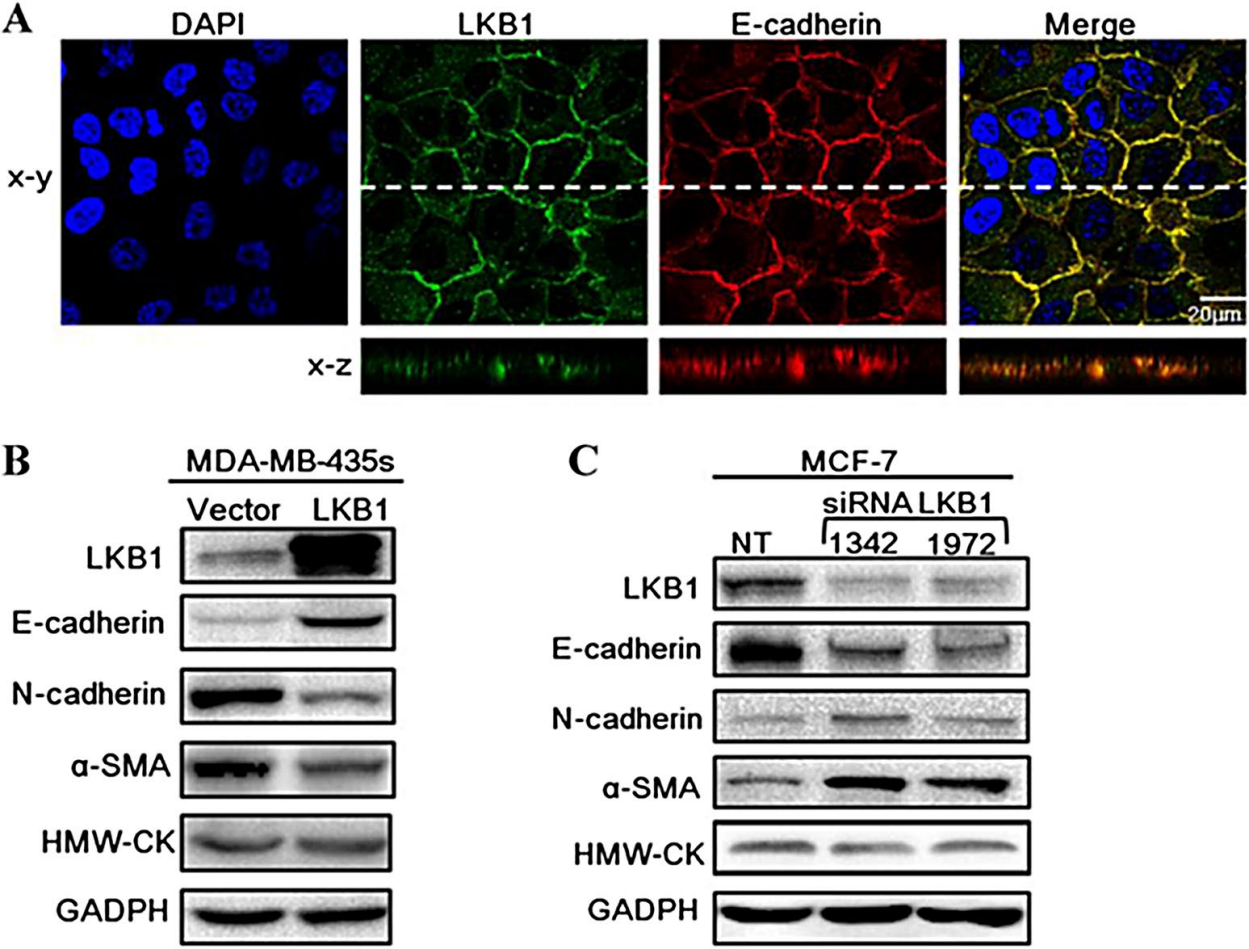
LKB1 was localized at adheren junctions and regulated the expressions of EMT markers. (**A**) MCF-10 A was stained for LKB1 (green), E-cadherin (red) and DAPI (blue). (**B**) Expressions of EMT markers in control and LKB1 overexpressing MDA-MB-435 s. (**C**) MCF-7 cells were transfected with non-targeting siRNA (NT) or siLKB1(1342/1972). The protein levels of E-cadherin, N-cadherin and α-SMA were determined by western blot

**Correct Figure 5**

**Fig. 5:**
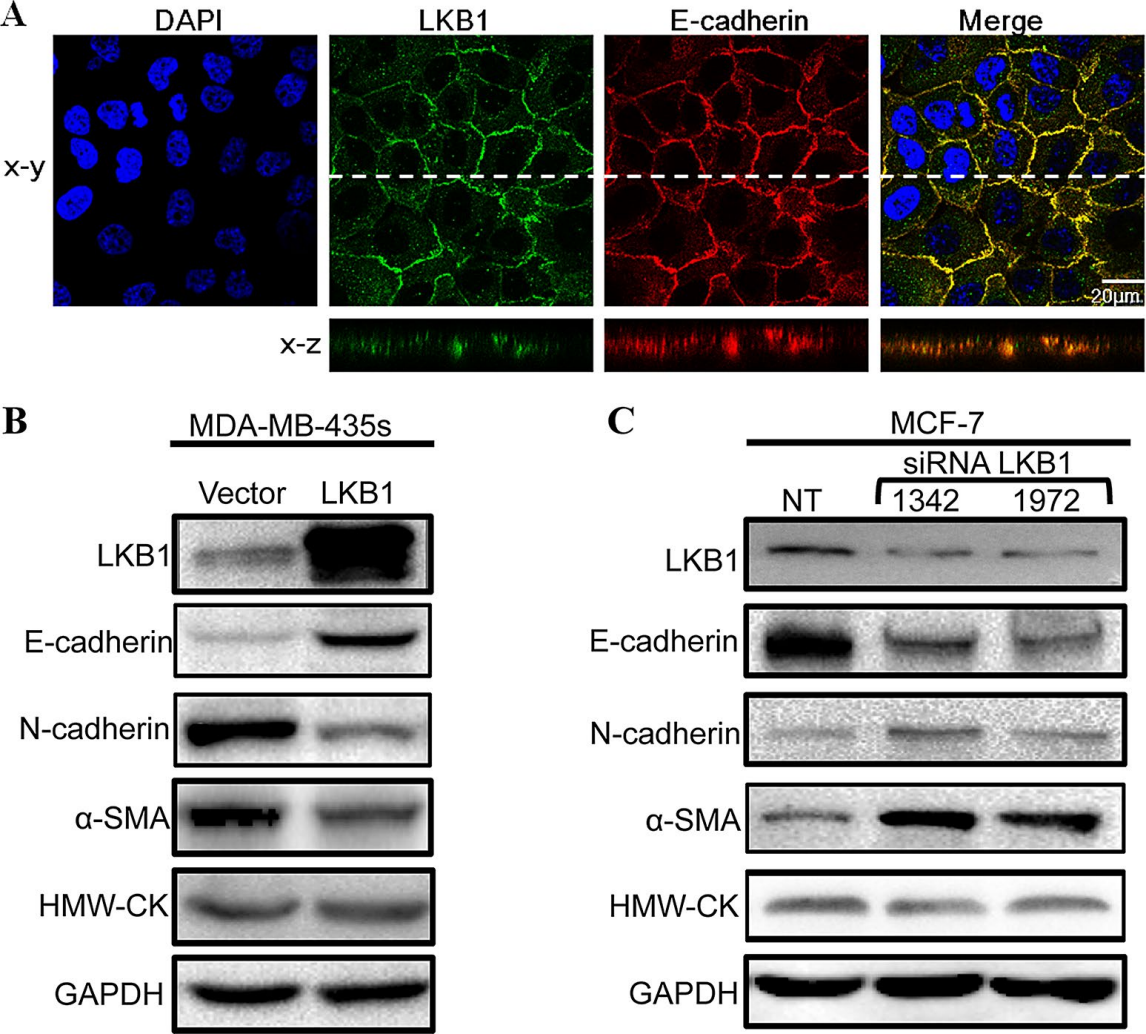
LKB1 was localized at adheren junctions and regulated the expressions of EMT markers. (**A**) MCF-10 A was stained for LKB1 (green), E-cadherin (red) and DAPI (blue). (**B**) Expressions of EMT markers in control and LKB1 overexpressing MDA-MB-435 s. (**C**) MCF-7 cells were transfected with non-targeting siRNA (NT) or siLKB1(1342/1972). The protein levels of E-cadherin, N-cadherin and α-SMA were determined by western blot
